# Cost-effectiveness of pre-exposure prophylaxis targeted to high-risk serodiscordant couples as a bridge to sustained ART use in Kampala, Uganda

**DOI:** 10.7448/IAS.18.4.20013

**Published:** 2015-07-20

**Authors:** Roger Ying, Monisha Sharma, Renee Heffron, Connie L Celum, Jared M Baeten, Elly Katabira, Nulu Bulya, Ruanne V Barnabas

**Affiliations:** 1 Department of Global Health, University of Washington, Seattle, WA, USA; 2 Department of Epidemiology, University of Washington, Seattle, WA, USA; 3 Department of Medicine, University of Washington, Seattle, WA, USA; 4 Infectious Disease Institute, College of Health Sciences, Makerere University, Kampala, Uganda; 5 Vaccine and Infectious Diseases Division, Fred Hutchinson Cancer Research Center, Seattle, WA, USA

**Keywords:** PrEP, mathematical modelling, serodiscordant couples, cost-effectiveness analysis, ART

## Abstract

**Introduction:**

Despite scale-up of antiretroviral therapy (ART) for treating HIV-positive persons, HIV incidence remains elevated among those at high risk such as persons in serodiscordant partnerships. Antiretrovirals taken by HIV-negative persons as pre-exposure prophylaxis (PrEP) has the potential to avert infections in individuals in serodiscordant partnerships. Evaluating the cost-effectiveness of implementing time-limited PrEP as a short-term bridge during the first six months of ART for the HIV-positive partner to prevent HIV transmission compared to increasing ART coverage is crucial to informing policy-makers considering PrEP implementation.

**Methods:**

To estimate the real world delivery costs of PrEP, we conducted micro-costing and time and motion analyses in an open-label prospective study of PrEP and ART delivery targeted to high-risk serodiscordant couples in Uganda (the Partners Demonstration Project). The cost (in USD, in 2012) of PrEP and ART for serodiscordant couples was assessed, with and without research components, in the study setting. Using Ministry of Health costs, the cost of PrEP and ART provision within a government programme was estimated, as was the cost of providing PrEP in addition to ART. We parameterized an HIV transmission model to estimate the health and economic impacts of 1) PrEP and ART targeted to high-risk serodiscordant couples in the context of current ART use and 2) increasing ART coverage to 55% of HIV-positive persons with CD4 ≤500 cells/µL without PrEP. The incremental cost-effectiveness ratios (ICERs) per HIV infection and disability-adjusted life year (DALY) averted were calculated over 10 years.

**Results:**

The annual cost of PrEP and ART delivery for serodiscordant couples was $1058 per couple in the study setting and $453 in the government setting. The portion of the programme cost due to PrEP was $408 and $92 per couple per year in the study and government settings, respectively. Over 10 years, a programme of PrEP and ART for high-risk serodiscordant couples was projected to avert 43% of HIV infections compared to current practice with an ICER of $1340 per infection averted. This was comparable to ART expansion alone, which would avert 37% of infections with an ICER of $1452.

**Conclusions:**

Using Uganda's gross domestic product per capita of $1681 as a threshold, PrEP and ART for high-risk persons have the potential for synergistic action and are cost-effective in preventing HIV infections in high prevalence settings. The annual cost of PrEP in this programme is less than $100 per serodiscordant couple if implemented in public clinics.

## Introduction

Antiretroviral therapy (ART) to treat HIV-positive persons has expanded to almost 10 million patients in low- and middle-income countries in 2013 [1, 2]. The increased coverage has led to significant health gains, such as an (95% confidence interval (CI): 9.6 to 12.9 years) increase in life expectancy by 11.3 years in KwaZulu-Natal, South Africa, from 2003 to 2011 [3]. Furthermore, ecologic data from Kwa Zulu-Natal indicate that ART is associated with substantial decreases in HIV incidence with a 38% (95% CI: 24–50%) reduction in HIV incidence associated with 30–40% ART coverage relative to <10% ART coverage [4]. However, despite the progress in treatment coverage, an additional 15 million HIV-positive persons who are eligible for ART have yet to start [2]. Thus, current coverage provides modest population-level reduction in HIV transmission. Expanding ART coverage may not be straightforward if asymptomatic persons with higher CD4 counts do not initiate ART and achieve durable viral suppression [5, 6]. Primary prevention strategies to prevent HIV acquisition are also needed particularly among high-risk persons. Antiretrovirals provided to HIV-negative persons as pre-exposure prophylaxis (PrEP) reduce the risk of HIV acquisition by up to 75% (95% CI: 55 to 87%) [7–9], and demonstration projects are currently underway to assess the real-world implementation of various PrEP strategies among target populations, including high-risk HIV serodiscordant couples [10, 11].

Populations such as those that participated in several PrEP trials face high annual risks of HIV acquisition [8, 12, 13], and targeting a package of biomedical and behavioural interventions, including PrEP, condoms, and HIV testing and counselling, to these populations may efficiently reduce HIV incidence [14, 15]. HIV serodiscordant couples represent a high-risk population among whom PrEP was shown to be effective [16]. For serodiscordant couples, the greatest period of risk for HIV transmission occurs when the HIV-positive partner is not virally suppressed, including times prior to and soon after initiating ART and during delayed or deferred therapy [5, 6]. During these high-risk periods, PrEP can be an integral component to a combination prevention strategy [17, 18].

Previous analyses found that targeting PrEP to high-risk groups is cost-effective [18–20]. However, these analyses vary widely in their assumptions about the cost of PrEP services [21]. No prior studies have conducted a micro-costing of the programmatic costs of PrEP implementation. To inform health economic analyses and optimize PrEP delivery strategies for HIV serodiscordant couples, micro-costing was conducted to estimate the additional operational costs of PrEP delivery in an open-label, prospective study. These data were then used in a mathematical model of HIV transmission in Kampala, Uganda, to project long-term health and economic outcomes and estimate cost-effectiveness of PrEP implementation.

## Methods

### Study clinic and intervention

The Partners Demonstration Project aims to assess the feasibility of antiretroviral-based interventions (ART and PrEP) to prevent HIV transmission among high-risk serodiscordant couples [22]. Short-term PrEP is used as a “bridge” to prevent transmission prior to viral suppression in the HIV-positive partner during periods where the HIV-positive partner has not yet initiated ART or may not be virally suppressed. Incremental costs were assessed for the programme as implemented at the Kasangati Health Centre – a peri-urban study clinic associated with the Infectious Diseases Institute approximately 15 km north of Kampala, Uganda.

Participants were recruited from local voluntary HIV testing and counselling clinics and community testing campaigns, and screened at Kasangati Health Centre. Eligible couples had HIV-positive partners that were not using ART, HIV-negative partners with normal renal function and a high level of HIV risk (assessed via a validated scoring tool) [23]. Briefly, the risk score is based on the characteristics of the HIV-negative partner (age and, if male, circumcision status) and the partnership (unprotected sex, number of children, and marriage or cohabitation status). Couples who scored ≥5 were considered to be at high risk and were invited to enrol while couples with lower scores were referred for care at a local HIV clinic. Upon enrolment, the HIV-negative partner was offered PrEP (co-formulated emtricitabine/tenofovir disoproxil fumarate (FTC/TDF)), and the couple received a comprehensive HIV prevention package including couples-based HIV prevention counselling and condoms. ART initiation (on site or via referral to a participant's clinic of choice) followed national treatment guidelines (CD4≤350 cells/µL before April 1, 2014; all HIV serodiscordant couples after April 1, 2014), and PrEP use was recommended until the HIV-positive partner had taken ART for at least six months. Couples returned for visits at one and three months after enrolment, and quarterly, thereafter.

### Clinic and participant characteristics

Enrolled individuals (*N*=292 couples) had a median age of 30 years, with over 95% of HIV-negative partners accepting PrEP and over 80% of HIV-positive partners initiating ART [11]. Participant retention was high with >85% of participants completing their expected visits [11] and over 80% of participants taking PrEP medication by blood tenofovir levels [24].

### Cost analysis

Data collection followed the Clinton Health Access Initiative guidelines for costing HIV interventions [25, 26] and the analysis was done from the payer/programmatic (Ministry of Health) perspective. Study budgets, government price lists and personnel interviews were used to estimate the start-up and recurrent costs of the intervention. Costs and activities were divided into three mutually exclusive categories: research, standard of care for couples counseling with ART delivery, and PrEP delivery. Research costs (e.g. completing informed consent) are excluded from this analysis. Standard of care costs and activities were those considered to be normal practice in couples-based HIV counselling and testing, such as sexual behaviour counselling, ART provision and adherence counselling for HIV-positive persons, and viral load monitoring 12 months after ART initiation. The remaining costs were considered PrEP costs (i.e. additional), and are the focus of this analysis. Time and motion studies were conducted to estimate the time needed to counsel participants for PrEP, the number of couples that could be seen annually and the allocation for joint costs. Data collection was done over three weeks from 20 January to 7 February 2014.

All costs and activities were divided into six mutually exclusive resource categories – start-up, personnel, medication, laboratory monitoring, transportation and building and supplies (see Supplementary Table 1). Data for start-up costs were collected from staff interviews and study budgets. Costs included interviewing, hiring and training staff; developing standard operating procedures; and recruiting participants. Personnel costs consisted of annual staff salaries collected from study budgets and weekly trainings that were directly observed (see Supplementary Table 2). Costs for antiretroviral medication (FTC/TDF/efavirenz (EFV) for treatment of HIV-positive partners, and FTC/TDF as PrEP for HIV-negative partners) were collected from drug price lists for the region. Costs of laboratory monitoring by external facilities and rapid diagnostic tests conducted at the health centre were acquired from invoices. Tests delivered to external facilities include CD4 and viral load measurements for the HIV-positive partner and serum creatinine for the HIV-negative partner. Rapid diagnostics conducted on-site include HIV and pregnancy tests. Transportation time and fuel costs incurred for delivering laboratory specimens were collected from interviews with drivers, reviews of driving records and receipts, and direct observation. Costs of buildings and supplies were collected from the market value of equivalent rental spaces and study budgets, respectively. All capital costs (e.g. vehicles and buildings) were annualized over five years with a discount of 3% [27, 28] and inflated to 2012 USD using Ugandan consumer price indices. Joint costs that required allocation were salaries, building, transportation and supplies.

To estimate clinic capacity, health staff were assumed to work an eight-hour workday for five days a week with a one hour break excluding national holidays and paid leave, in line with Ugandan labour guidelines [29] (see Supplementary Table 3). The amount of time per visit type (screening, enrolment, follow-up) was used to estimate the total number of couples the clinic could enrol in 12 months, assuming a screen-to-enrol ratio of 73% and 12-month retention of 97% as observed during the study follow-up period.

### Ministry of health scenario

To estimate the Ministry of Health cost for a PrEP programme, costs and procedures were revised to reflect a programme that would be implemented by the government. First, private-sector salaries were replaced with public-sector salaries using 2009 estimates adjusted to 2012 using the ratio of Uganda's consumer price indices in 2012 and 2009 [30]. Second, the annual cost of PrEP medication (FTC/TDF) was reduced from current Ugandan private-sector list price ($382) to the lowest estimate as negotiated by the Clinton Health Access Initiative ($75) [31]. Third, viral load tests were assumed to occur only at Month 12 for clinical monitoring, with laboratory tests for viral load and HBV screening replaced with point-of-care tests ($20 and $0.50, respectively) [32, 33]. Finally, a previously validated model of task-shifting used by Médecins Sans Frontières that allowed nurses to prescribe ART, used adherence counsellors and organized HIV support groups [34] was used to estimate the impact of task-shifting on clinic capacity. The task-shifting programme did not impair treatment outcomes and improved ART adherence.

### Cost-effectiveness analysis

The estimated Ministry of Health incremental cost for a PrEP programme was incorporated into a dynamic transmission model of HIV [35] that was parameterized to southwest Uganda using estimates from the literature and a study of home HIV testing and counselling in southwest Uganda [36]. The model is stratified by age, gender and sexual activity, and includes HIV stage by CD4 T-cell count and HIV RNA viral load, and was calibrated to fit HIV incidence and prevalence from the region. Further details can be found in Supplementary Table 4 and Supplementary file 2. The model was used to estimate the cost-effectiveness of 1) implementing a PrEP and ART programme for high-risk serodiscordant couple, or 2) scaling-up ART to the new ART initiation guidelines (CD4≤500 cells/µL). The incremental cost-effectiveness ratios (ICERs) were calculated for each scenario.

The first scenario simulated current ART coverage in Uganda of 40% among all HIV-positive persons, with 60% coverage for persons with CD4 ≤200 cells/µL, 50% for persons with CD4 200–350 cells/µL and 10% for persons with CD4 350–500 cells/µL. The second scenario simulated increased ART coverage for persons with CD4 ≤500 cells/µL as the recently changed ART eligibility criteria are implemented, such that ART coverage for persons with CD4 350–500 cells/µL is 50%, assuming the same coverage of ART as seen with previous ART initiation guidelines. The third scenario simulated PrEP and ART targeted to 90% of high-risk serodiscordant couples. In the model, high-risk serodiscordant couples are defined as those partnerships in which the HIV-negative partner is aged <25 years and belongs to the high sexual activity group (i.e. the top 15th percentile in the number of casual sex partners). Annual drop-out rates from ART and PrEP were assumed to be 6% [37].

To estimate the health and economic impact of the modelled scenarios, costs of PrEP delivery, ART [25] and hospitalization [38] were used to calculate the ICER per HIV infection averted and disability-adjusted life year (DALY) averted for each scenario (ART scale-up or PrEP and ART for high-risk serodiscordant couples). The ICER was calculated using outcomes from a 10-year time horizon, with costs and effectiveness measures discounted by 3% annually. Consistent with health-economic conventions [27], we regard an intervention as very cost-effective if the cost per DALY or HIV infection averted is less than Uganda's per capita gross domestic product (GDP) in 2012 ($1681) [39], and cost-effective if the cost per DALY averted is less than three times Uganda's per capita GDP ($5043).

### Sensitivity analyses

To explore how the ICER of the PrEP programme changes with different programmatic assumptions, sensitivity analyses were conducted. First, the clinic capacity was varied from 200 to 1500 couples retained for 12 months, assuming a constant number of staff and 97% patient retention over 12 months. Second, the cost of PrEP delivery based on clinic capacity (200 to 1500 couples retained at Month 12), efficacies of ART and PrEP for reducing HIV transmission (73 to 99% [40] and 77 to 98% [41], respectively), drop-out rate from ART and PrEP (0 to 10%), annual discount rate (0 to 10%) and ART cost ($100 to $500 per person per year) were all varied independently to estimate the sensitivity of the ICER.

## Results

### Overall and PrEP intervention costs

Seven screening visits, five enrolment visits, and eighteen follow-up visits were observed. The average total visit times including research components for the screening, enrolment and follow-up visits were 2.7, 3.8 and 1.3 hours, respectively. As studied, the cost of PrEP components, incremental to the cost of ART for serodiscordant couples, was $408 annually per couple. In the best-case scenario using Ministry of Health prices, the incremental cost of PrEP components decreased to $92 annually per couple.

### “As Studied” scenario

**Figure 1:**
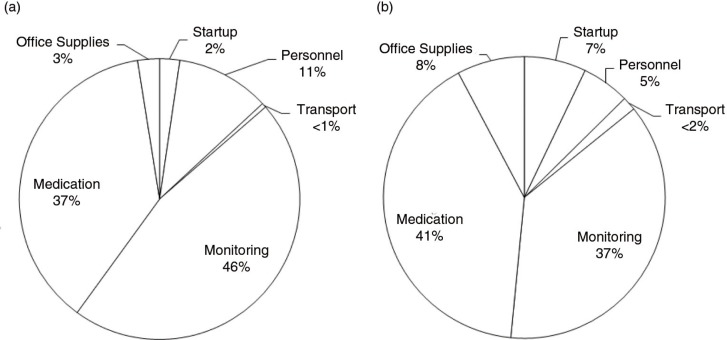
Additional PrEP programme costs by resource type. The allocation of costs by resource type for the intervention “As Studied” (a) and in the “Ministry of Health” (b). “Ministry of Health” includes public-sector salaries, fewer laboratory tests, less expensive medication and task-shifting.

In the “As Studied” estimate of $408 annually per couple, we assumed that four nurse counsellors and two clinicians provided care in a clinic that could screen 1086 couples per year, enrol 793 (73%) of them and retain 769 (97%) by Month 12 (Table 1). This programme would cost $827,351 ($1058 per couple retained for 12 months), with the majority of the costs going towards medication and laboratory monitoring (54 and 26%, respectively). Of the total cost, 44% ($363,012) was attributable to the PrEP programme and 56% ($464,340) to standard of care. Considering only the additional costs of PrEP (Figure 1a), PrEP-related laboratory monitoring costs contribute 46% of additional PrEP intervention costs, whereas PrEP medication costs contribute 37% of additional PrEP intervention costs.

**Table 1:** Comparison of outcomes excluding research components

		Time per visit (hours)				
				
Scenario		Screening	Enrolment	Follow-up	No. of couples at Month 12	Cost per couple
As studied^a^	Total clinical^c^	1.5	2.5	1.1	769	$1058
	PrEP	0.6	1.3	0.6		$408
Ministry of Health^b^	Total clinical^c^	1.4	1.5	0.7	1111	$453
	PrEP	0.4	0.6	0.4		$92

The time per visit was estimated from time and motion observations at the clinic

a outcomes as observed in the Partners Demonstration Project

b assumes public-sector salaries, point-of-care laboratory tests, less expensive medication and task-shifting

c includes standard care and PrEP components.

### Ministry of health scenario

The Ministry of Health scenario assumes that PrEP delivery by the government would cost less than as implemented in the study as salaries and medication costs would be lower than those in the research setting. In addition, less laboratory monitoring would be conducted, according to national guidelines (Table 2).

**Table 2:** Change in costs with additional assumptions

Programme change	Number of couples	Total cost per couple	ART only cost per couple	Additional PrEP cost per couple
Baseline (No PrEP)	769	$650	$650	$0
As Studied with PrEP	769	$1058	$650	$408
With public-sector staff salaries	769	$1005	$635	$370
With reduced medication cost	769	$720	$466	$254
With fewer laboratory tests^a^	769	$497	$396	$101
With task-shifting	1111	$453	$361	$92

The impact of programmatic changes on the capacity of a PrEP programme and the annual cost per couple retained for one year

a simplified testing with one point-of-care HBV test and one point-of-care viral load measurement.

With public-sector salaries, the additional cost per couple decreased from $408 to $370 annually. Using the annual per-person cost of PrEP (FTC/TDF) negotiated by the Clinton Health Access Initiative ($75) [31], the annual additional cost of intervention per couple decreased from $370 to $254, and reduced the medication portion of the total cost from 41 to 15%. Using one point-of-care viral load test conducted at 12 months to monitor clinical response to ART and one point-of-care HBV test to screen HIV-negative partners, the annual cost per couple decreased from $254 to $101. Finally, task-shifting of clinical activities resulted in screening, enrolment and follow-up visit times of 1.4, 1.5 and 0.7 hours, respectively (Table 1), and increased clinic capacity to 1111 couples retained at Month 12. As a result, the annual additional cost per couple decreases to $92. In the Ministry of Health scenario, the proportion of costs due to laboratory monitoring decreased from 46% in the study to 37%, whereas the proportion of costs due to medication increased from 37% in the study to 41% (Figure 1b).

### Cost-effectiveness analysis

In the cost-effectiveness analysis (Table 3), targeting PrEP and ART to high-risk serodiscordant couples averts 43% more HIV infections than baseline and is cost-effective with an ICER of $1340 per HIV infection averted over 10 years, whereas ART scale-up alone averts 37% more HIV infections than baseline, costing $1452 per incident HIV case averted relative to baseline.

**Table 3:** Incremental cost-effectiveness ratios (ICERs) of ART and PrEP strategies for southwest Uganda

Outcome	Scenario	Effectiveness	Cost (millions USD)	ICER
HIV infections averted	Baseline: Current ART uptake ART: Baseline (40%)^a^ PrEP: N/A	94,000	185	Baseline
	ART scale up only (no PrEP) ART: CD4≤500 cells/µL (55%) PrEP: N/A	104,000 (37%)	200	Dominated^b^
	MoH adds PrEP programme for all high-risk serodiscordant couples ART: Baseline (40%)^a^+high-risk couples^c^ without CD4/VL criteria (80%) PrEP: High-risk couples^c^ (80%)	120,000 (43%)	219	$1340
DALYs averted	Baseline: Current ART uptake ART: Baseline (40%)^a^ PrEP: N/A	203,000	185	Baseline
	ART scale up only (no PrEP) ART: CD4≤500 cells/µL (55%) PrEP: N/A	217,000 (60%)	200	$1075
	MoH adds PrEP programme for all high-risk serodiscordant couples ART: Baseline (40%)^a^+high-risk SDC^c^ without CD4/VL criteria (80%) PrEP: High-risk couples^c^ (80%)	221,000 (62%)	219	$5354

Results are shown for a 10-year time horizon relative to 2014

a under former guidelines

b extended dominance occurs when a strategy is less cost-effective than a combination of other strategies

c high-risk serodiscordant couples are those in which the HIV-negative partner is ≤25 years old and both partners are in the top 15th percentile in the number of casual sexual partners.

When considering the outcome of DALYs averted, the ICER for PrEP and ART together is higher than for ART scale-up alone. It is the most effective strategy, averting 62% more DALYs than baseline, but the ICER of $5354 per DALY averted is slightly higher than three times Uganda's GDP per capita ($5043), the threshold for cost-effectiveness. In this case, scaling up ART only was the most cost-effective strategy at $1075 per DALY averted while averting 60% more DALYs than baseline.

### Sensitivity analysis

**Figure 2:**
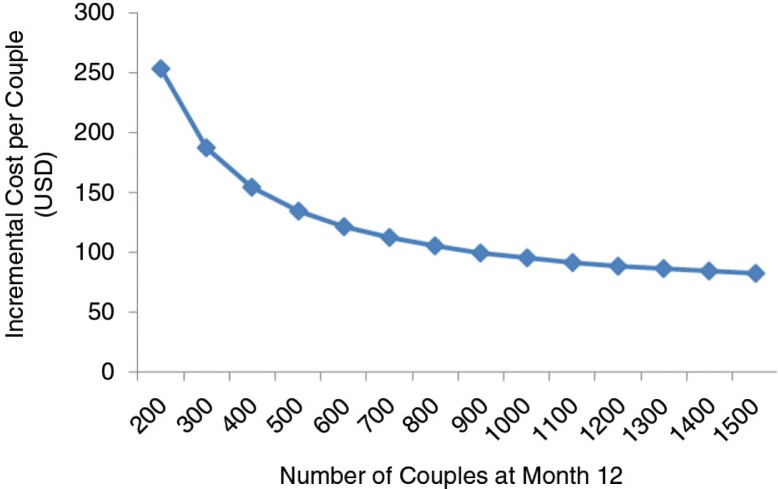
Annual incremental cost per couple by annual number of couples enrolled. The costs are based on the Ministry of Health scenario with public-sector salaries, fewer laboratory tests, less expensive medication and task-shifting. The clinic capacity assumes a screen-to-enrol ratio of 1.37, and 97% retention of enrolled couples over 12 months.

Increasing the clinic capacity from 200 to 1500 couples annually in the primary cost estimates from Table 1 decreased the additional cost per couple in the PrEP programme at Month 12 from $254 to $82 (Figure 2), suggesting that the incremental cost can increase substantially if clinic capacity is very low.

**Figure 3:**
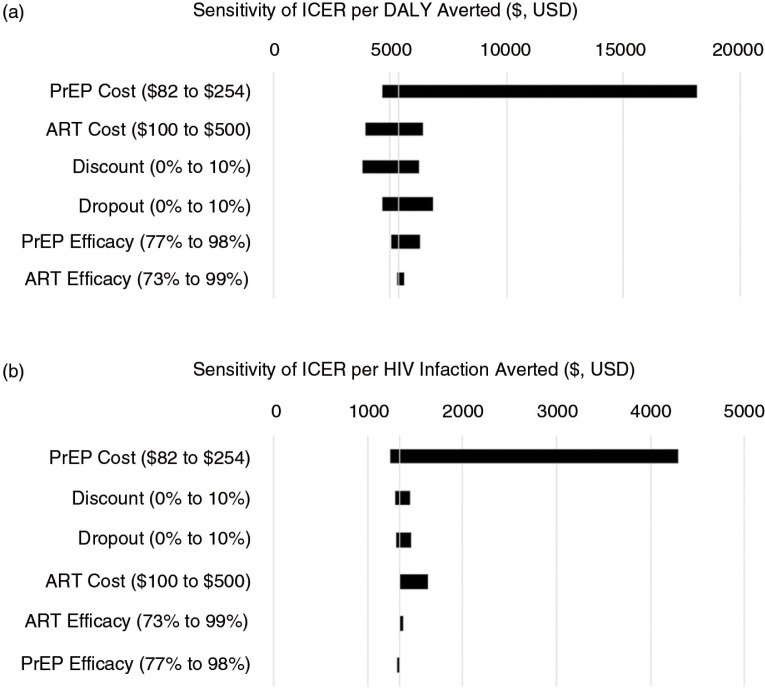
Sensitivity of the ICER per DALY (a) and HIV infection (b) averted for the high-risk serodiscordant couples PrEP programme. The base case ICER is $5354 per DALY averted and $1340 per HIV infection averted.

In sensitivity analyses, a high clinic capacity (1500 couples annually, costing $82 per couple) reduced the ICER of the PrEP programme to $4648 per DALY averted, whereas low clinic capacity (200 couples annually, costing $254 per couple) increased the ICER to $18,151 per DALY averted. Similarly, the cost per HIV infection averted increases dramatically with decreased clinic capacity. With ART cost at $100 per person per year, no annual discounting and 10% drop-out from ART and PrEP, the PrEP programme becomes cost-effective for averting DALYs, although the programme never becomes very cost-effective for averting DALYs. For averting HIV infections, PrEP remains the most cost-effective strategy across all ranges of assumptions. It is consistently very cost-effective (i.e. less than Uganda's per capita GDP) except when assuming low per person annual ART cost ($100), in which the ICER per infection averted is $521 for ART scale-up and $1515 for the PrEP programme (Figure 3).

## Discussion

This comprehensive micro-costing of implementing PrEP as a “bridge” among high-risk serodiscordant couples until ART initiation and viral suppression by the HIV-positive partner was used to inform a dynamic simulation model of HIV transmission. Adding a PrEP bridging intervention until ART initiation to standard of care by WHO and Ugandan guidelines is very cost-effective for averting HIV infections in generalized HIV epidemic settings, such as Kampala, Uganda. The average private sector “as studied” clinical cost per couple retained after 12 months was $1058, with about half of the cost ($408) being due to the PrEP intervention. The majority (83%) of the PrEP costs were attributable to laboratory monitoring and PrEP medication (FTC/TDF). If this programme were implemented by the Ministry of Health, government salaries, reduced drug costs, fewer laboratory tests and task-shifting could reduce cost and increase efficiency, resulting in a PrEP intervention cost of less than $100 per couple per year. Comparing the ICERs for a PrEP and ART programme targeted to serodiscordant couples, and expanding ART coverage relative to current practice, could guide expansion of HIV prevention programmes. For averting HIV infections, implementing a PrEP and ART programme for high-risk serodiscordant couples is very cost-effective, and increasing ART coverage to 55% of HIV-positive persons with CD4≤500 cells/µL without PrEP is not cost-effective. When the outcome considered was DALYs averted, PrEP and ART together averted the most DALYs but slightly exceeded the cost-effectiveness threshold of three times Uganda's GDP per capita. Instead, increasing ART coverage is the most cost-effective strategy. These results are explained by PrEP being an HIV prevention intervention, and the majority of the intervention's impact on averting DALYs due to HIV not being captured within the 10-year time horizon of the analysis. ART treatment alone, in contrast, has an immediate effect on averting DALYs, particularly for those at lower CD4 counts.

Reaching efficient PrEP implementation will require clearly defined strategies for intervention delivery. The WHO recently published guidelines that recommended targeting PrEP to serodiscordant couples, and men and transgender women who have sex with men [42] and added that demonstration projects are necessary to develop reasonable frameworks for delivering PrEP. Initial studies of the impact of PrEP as a bridge to ART among serodiscordant couples estimate a 96% reduction in HIV incidence [11]. Further studies are needed to verify the assumptions made to achieve efficient scenarios that maximize demand and clinic capacity. Efficient scale-up will depend on increased patient and provider knowledge of PrEP, as well as increased accessibility of PrEP drugs with couples counselling. Finally, task-shifting has been piloted in several regions in sub-Saharan Africa and results show that shifting clinical responsibilities from physicians to other staff does not necessarily affect clinical outcomes [43] and, in some cases, may improve them [44]. Although we estimated that task-shifting reduced the amount of time per clinic visit compared to the demonstration project by more efficiently using staff skills, the overall impact on cost per couple is small.

This analysis has several limitations. We assume that there is sufficient demand for PrEP services such that the clinic is at full capacity. Staff likely have other health care tasks unrelated to PrEP delivery, but data to quantify this were not available. In addition, the act of observing counselling sessions may influence the counselling interaction. However, clinic staff were informed that observations were related to a costing analysis and not a staff evaluation, and multiple staff were observed over the three-week period to ensure robustness of the data. Moreover, study staff had more training and experience in couples counselling and PrEP provision than is typical at public health clinics. However, the sensitivity analysis suggests that even if fewer couples were retained at Month 12 and the screening-to-enrolment ratio were higher, the cost per couple would not change substantially, though the ICER would increase dramatically at very low clinic capacities. Our model also makes assumptions regarding ART uptake under new guidelines (CD4≤500 cells/µL), which we conservatively assume as 55% of all HIV-positive persons achieving viral suppression, assuming ART uptake among asymptomatic persons is the same as was seen among symptomatic individuals. While this assumption is realistic in the short term, it favourably impacts our assessment of PrEP cost-effectiveness. Increasing the coverage and adherence to ART would result in a lower cost-effectiveness per HIV infection averted for a combined PrEP and ART programme. However, data on ART adherence under the new guidelines are insufficient.

To our knowledge, these are the first primary cost estimates for PrEP counselling and provision in Africa. Previous studies have used lower cost estimates for HIV testing and counselling than found here in the Partners Demonstration Project, leading their estimates to be between our “as studied” and “Ministry of Health” scenarios [45–47]. Previous modelling studies of PrEP focusing on South Africa have found PrEP to cost less than two times the per capita GDP per HIV infection averted [21], similar to our estimate, but the estimates are not directly comparable due to differences in the HIV epidemics.

## Conclusions

ART coverage in sub-Saharan Africa has been rapid and successful, but only approximately one-third of HIV-positive persons are virally suppressed. Additional interventions are needed to give individuals at high risk of HIV acquisition a method for protecting themselves. PrEP can serve as a short-term primary prevention strategy during periods of high risk [48]. This analysis suggests that incorporating PrEP into existing HIV testing and counselling and ART programmes is a cost-effective method for HIV prevention.

## Supplementary Material

Cost-effectiveness of pre-exposure prophylaxis targeted to high-risk serodiscordant couples as a bridge to sustained ART use in Kampala, Uganda

Cost-effectiveness of pre-exposure prophylaxis targeted to high-risk serodiscordant couples as a bridge to sustained ART use in Kampala, Uganda
