# Supplementary material for: Cost-effectiveness of pre-exposure prophylaxis targeted to high-risk serodiscordant couples as a bridge to sustained ART use in Kampala, Uganda
Source: J Int AIDS Soc. 2015 Jul 20;18(4Suppl 3):20013. doi: 10.7448/IAS.18.4.20013 (PMC4509901; doi:10.7448/IAS.18.4.20013)
Supplement: Cost-effectiveness of pre-exposure prophylaxis targeted to high-risk serodiscordant couples as a bridge to sustained ART use in Kampala, Uganda [file JIAS-18-20013-S001.pdf]

Appendix

Pre-exposure prophylaxis targeted to high-risk serodiscordant couples as a bridge to sustained ART use has the potential to cost-effectively reduce HIV incidence in Kampala, Uganda

Table 1. Total Costs by Resource Type and Allocation.

Resource Type	As Studied		Ministry of Health	
	**Current Care**	**PrEP**	**Current Care**	**PrEP**
Start-up	$0	$7,273	$0	$7,273
Personnel	$36,043	$33,877	$8,367	$5,626
Transport	$1,607	$1,607	$1,607	$1,607
Laboratory Monitoring	$23,913	$195,004	$34,560	$38,239
Medication	$324,142	$117,614	$280,583	$41,665
Office Supplies	$71,363	$7,929	$71,363	$7,929
**Total**	**$457,068**	**$363,304**	**$396,480**	**$102,339**

Table 2. Cost Assumptions in Study and Ministry of Health Setting. Staff salaries in Ministry of Health are adjusted from 2009 using Ugandan CPI.

	**Value in Study**		**Value in Ministry of Health**	
**Staff Position**	**Annual Salary (USD)**	**No. of Staff**	**Annual Salary (USD) [1]**	**No. of Staff**
Administrator	$30,000	0.7	$5,496	1
Study Coordinator	$15,000	0.05	$4,263	0
Clinician	$15,000	1	$5,496	0.05
Nurse Counselor	$10,000	1	$3,926	1
Counselor	$10,000	3.6	$1,823	4
Community Recruiter	$10,000	2.8	$1,823	0.5
Pharmacist	$10,000	1.1	$1,823	1
Laboratory Technician	$10,000	1	$1,823	1
Data Manager	$10,000	4	$1,823	0
Driver	$7,000	3	$1,085	1
**Laboratory Test**	**Cost per Test (USD)**	**Tests per Year**	**Cost per Test (USD)**	**Tests per Year**
Viral Load Test	$63.25	3	$20	2
HBV Test	$25	1	$0.50	1
CD4 Test	$9	3	$9	3
Serum Creatinine	$8.50	2	$8.50	2
**Medication**	**Annual Per-Person Cost (USD)**		**Annual Per-Person Cost (USD)**	
TDF-FTC-EFV	$382		$156 [2]	
TDF-FTC	$382		$75 [2]	

Table 3. Staffing and Clinic Assumptions.

Clinic Attribute	Value
**Days worked per year**	218 days
	(assumes 2.5 vacation days per month and 13 national holidays)
**Staff Training**	24 hours
	(assumes two hours per week for three months)
**Screening : Enrollment**	1.37
**Annual Drop-out**	3%
**Visits per Couple**	7

Table 4. Key parameters used in model. The parameters were based on the Home HTC study and other literature. For parameters with varying estimates, we chose values that best fit our data.

Model Parameter	Value [Range]	Reference
**Duration of Disease**		
**By CD4 Count**		
Acute	0.25 year [0.2, 0.25]	Johnson *et al.* [3]
>500 cells/µL	1.88 years	Celum *et al.*, Baeten *et al.* [4, 5]
500 to 350 cells/µL	1.22 years	Celum *et al.*, Baeten *et al.* [4, 5]
350 to 200 cells/µL	5.90 years	Celum *et al.*, Baeten *et al.* [4, 5]
≤200 cells/µL	1.96 years (95% CI: 3.0-4.3 years)	Badri *et al.* [6]
**By HIV Viral Load**		
Acute	0.25 year	Johnson *et al.* [3]
<1,000 copies/mL	3.13 years	Celum *et al.*, Baeten *et al.* [4, 5]
1,000-10,000 copies/mL	1.99 years	Celum *et al.*, Baeten *et al.* [4, 5]
10,000-50,000 copies/mL	4.40 years	Celum *et al.*, Baeten *et al.* [4, 5]
>50,000 copies/mL	1.44 years	Estimated
**Transmission Probability***		
Baseline Probability	0.0006	Boily *et al.*, Powers *et al.* [7, 8]
Acute	26 x Baseline [17.21, 38.27]	Hollingsworth *et al.* [9]
VL≤1,000 copies/mL	1 x Baseline [0.01, 11]	Quinn *et al.*
VL 1,000-10,000 copies/mL	5.8 x Baseline [2.26, 17.80]	Quinn *et al.* [10]
VL 10,000-50,000 copies/mL	6.9 x Baseline [2.96, 20.15]	Quinn *et al.* [10]
VL>50,000 copies/mL	11.9 x Baseline [5.02, 34.88]	Quinn *et al.* [10]
**Intervention Efficacy for Reducing HIV Transmission**		
Antiretroviral therapy (ART)	96% [0.73, 0.99]	Cohen *et al.* [11]
Pre-exposure prophylaxis (PrEP)	92% [0.77, 0.98]	Baeten *et al.* [5]
**Baseline ART Coverage**		
Before 2000	0%	Windisch *et al.* [12]
2013	40% of all HIV-positive persons	Barnabas *et al.* [13]
**Prevalence of Circumcision**		
**2012**	30%	Uganda AIS [14]
**Costs‡**		
ART	$269 per person per year	CHAI [15]
Hospitalization: pre-ART CD4≤200 cells/µL	$27.30 per HIV-positive person per year	Meyer-Rath *et al.* [16]
Hospitalization: pre-ART CD4 200-350 cells/µL	$13.08 per HIV-positive person per year	Meyer-Rath *et al.* [16]
Hospitalization: pre-ART CD4>350 cells/µL	$8.80 per HIV-positive person per year	Meyer-Rath *et al.* [16]
Hospitalization: post-ART CD4 200-350 cells/µL	$25.04 per HIV-positive person per year	Meyer-Rath *et al.* [16]
Hospitalization: post-ART CD4>350 cells/µL	$10.15 per HIV-positive person per year	Meyer-Rath *et al.* [16]

*Probability of HIV transmission per coital act assumes that HIV transmission is a Bernoulli process.
‡Extrapolated and calibrated using country GDP.

Table 5. Utility weights for estimating disability-adjusted life-years averted.

Health State	DALY Weight [17]
**HIV-negative**	**0**
**HIV-positive CD4>350**	**0.053**
**HIV-positive CD4 200-350**	**0.221**
**HIV-positive CD4<200**	**0.547**
**HIV-positive on ART**	**0.053**
**Dead**	**1**

References

1. Africa Health Workforce Observatory. Human Resources for Health: Country Profile, Uganda. In: WHO; 2009.
2. Antiretroviral (ARV) Ceiling Price List. In: The Clinton Health Access Initiative; 2013.
3. Johnson L, Dorrington R, Bradshaw D, Van Wyk V, Rehle T. Sexual behaviour patterns in South Africa and their association with the spread of HIV: Insights from a mathematical model. *Demographic Research* 2009,**21**:289 - 340.
4. Celum C, Wald A, Lingappa JR, Magaret AS, Wang RS, Mugo N*, et al.* Acyclovir and transmission of HIV-1 from persons infected with HIV-1 and HSV-2. *N Engl J Med* 2010,**362**:427-439.
5. Baeten JM, Donnell D, Ndase P, Mugo NR, Campbell JD, Wangisi J*, et al.* Antiretroviral prophylaxis for HIV prevention in heterosexual men and women. *N Engl J Med* 2012,**367**:399-410.
6. Badri M, Bekker LG, Orrell C, Pitt J, Cilliers F, Wood R. Initiating highly active antiretroviral therapy in sub-Saharan Africa: an assessment of the revised World Health Organization scaling-up guidelines. *AIDS* 2004,**18**:1159-1168.
7. Boily MC, Baggaley RF, Wang L, Masse B, White RG, Hayes RJ*, et al.* Heterosexual risk of HIV-1 infection per sexual act: systematic review and meta-analysis of observational studies. *Lancet Infect Dis* 2009,**9**:118-129.
8. Powers KA, Poole C, Pettifor AE, Cohen MS. Rethinking the heterosexual infectivity of HIV-1: a systematic review and meta-analysis. *Lancet Infect Dis* 2008,**8**:553-563.
9. Hollingsworth TD, Anderson RM, Fraser C. HIV-1 transmission, by stage of infection. *J Infect Dis* 2008,**198**:687-693.
10. Quinn TC, Wawer MJ, Sewankambo N, Serwadda D, Li C, Wabwire-Mangen F*, et al.* Viral load and heterosexual transmission of human immunodeficiency virus type 1. Rakai Project Study Group. *N Engl J Med* 2000,**342**:921-929.
11. Cohen MS, Chen YQ, McCauley M, Gamble T, Hosseinipour MC, Kumarasamy N*, et al.* Prevention of HIV-1 infection with early antiretroviral therapy. *N Engl J Med* 2011,**365**:493-505.
12. Windisch R, Waiswa P, Neuhann F, Scheibe F, de Savigny D. Scaling up antiretroviral therapy in Uganda: using supply chain management to appraise health systems strengthening. *Global Health* 2011,**7**:25.
13. Barnabas RV, Van Rooyen H, Baeten J, Tumwesigye E, Phakathi Z, Tumwebaze H*, et al.* High testing uptake and linkages to HIV treatment through home-based HIV counseling and testing and facilitated referral in Kabwohe, Uganda and KwaZulu-Natal (KZN), South Africa. In: *HIV Treatment as Prevention Workshop*. Vancouver, Canada; 2012.
14. International UMoHaI. 2011 Uganda AIDS Indicator Survey: Key Findings. In. Maryland, USA: MOH and ICF International; 2012.
15. Multi-Country Analysis of Treatment Costs for HIV/AIDS (MATCH): Unit costing at 161 Representative Facilities in Ethiopia, Malawi, Rwanda, South Africa and Zambia. In; 2012.
16. Meyer-Rath G, Brennan AT, Fox MP, Modisenyane T, Tshabangu N, Mohapi L*, et al.* Rates and cost of hospitalization before and after initiation of antiretroviral therapy in urban and rural settings in South Africa. *J Acquir Immune Defic Syndr* 2013,**62**:322-328.
17. Salomon JA, Vos T, Hogan DR, Gagnon M, Naghavi M, Mokdad A*, et al.* Common values in assessing health outcomes from disease and injury: disability weights measurement study for the Global Burden of Disease Study 2010. *Lancet* 2012,**380**:2129-2143.
