# Supplementary material for: Cost-effectiveness of pre-exposure prophylaxis targeted to high-risk serodiscordant couples as a bridge to sustained ART use in Kampala, Uganda
Source: J Int AIDS Soc. 2015 Jul 20;18(4Suppl 3):20013. doi: 10.7448/IAS.18.4.20013 (PMC4509901; doi:10.7448/IAS.18.4.20013)
Supplement: Cost-effectiveness of pre-exposure prophylaxis targeted to high-risk serodiscordant couples as a bridge to sustained ART use in Kampala, Uganda [file JIAS-18-20013-S002.pdf]

Model specifications for

Cost-effectiveness of pre-exposure prophylaxis targeted to high-risk serodiscordant couples as a bridge to sustained ART use in Kampala, Uganda

I. Technical Specifications
II. Interventions
III. Epidemiological Parameters
IV. References

General Overview:

The purpose of this mathematical model is to study the impact of antiretroviral therapy (ART) provision to HIV-positive persons in a generalized HIV epidemic setting. We created a model that simulates heterosexual HIV transmission and is parameterized to the HIV epidemic in Kampala, Uganda. The model reproduces population-level dynamics and stratifies the population by age, gender, and sexual risk. We structure the model to examine using home-based HIV testing and counseling campaigns to scale-up ART targeted to HIV-positive persons by CD4+ T-cell (CD4) count and HIV RNA concentration (viral load).

The model begins with an entirely HIV-negative population at time $t = 0$ with a size reflecting Kampala in 1970 and age distribution reflecting Uganda in 1980 [1]. In the first iteration ($t =$ 1970), 0.01% of the population is HIV-positive, and subsequent iterations evaluate the demography, enrollment, and aging of the population using 4th-order Runge-Kutta methods. The population dynamics are governed by a system of ordinary differential equations (ODEs) that are solved in MATLAB. The model iterates in three-month intervals from 1970 to 2030.

I. Technical Specifications

HIV Natural History:

The natural history of HIV infection is modeled in stages defined by CD4 count and viral load as shown in Figure S1. When a person becomes HIV-infected, s/he enters the acute stage characterized by a short duration and high probability of HIV transmission. The person then progresses through stages of CD4 count and viral load at rates ν^d and ω^v, respectively, where d represents the current CD4 count and v represents the current viral load. The parameters ν^d and ω^v are based on an analysis of disease progression using data from the Partners HSV/HIV and

Partners PrEP studies. The average life expectancy from infection to death for untreated persons is 10.7 years.

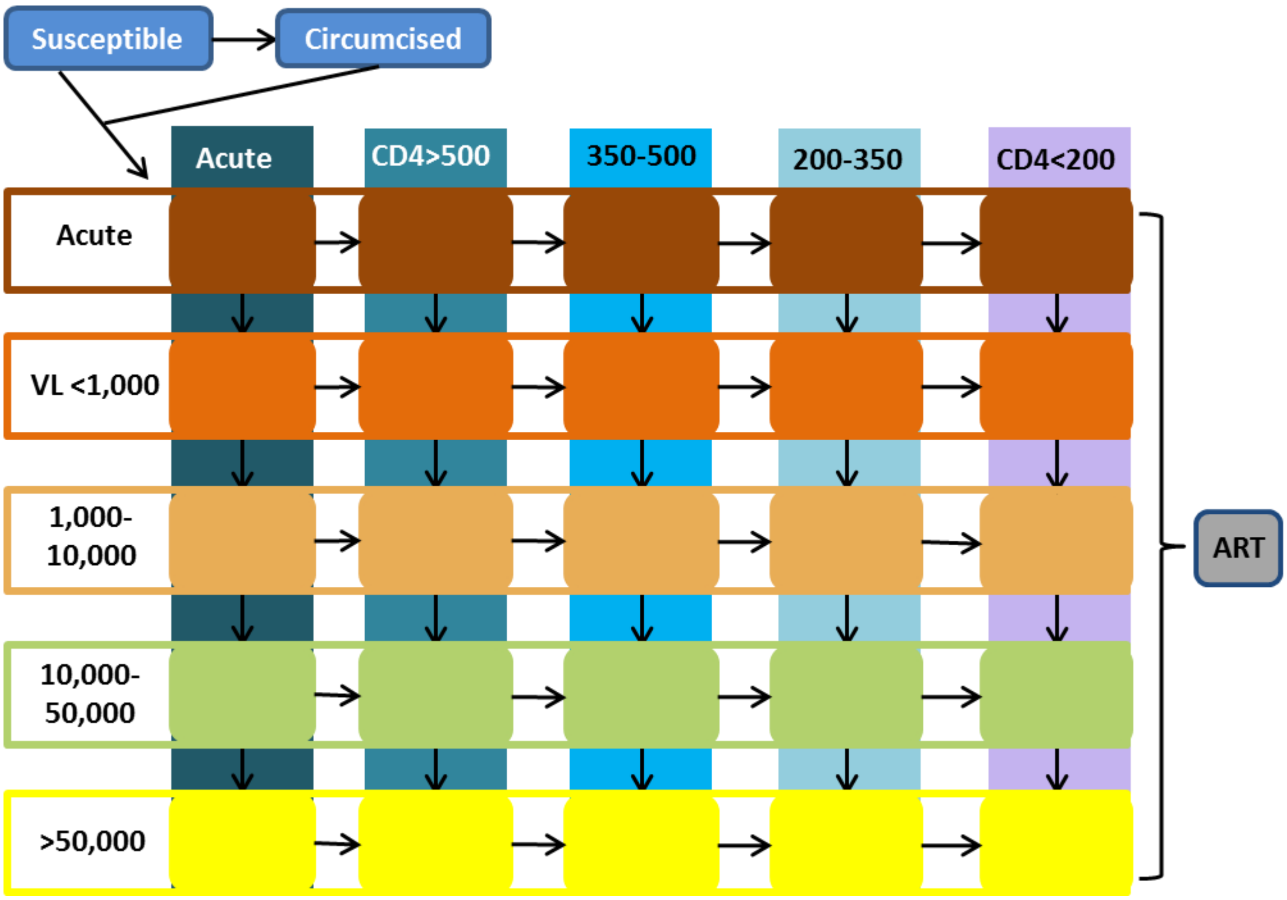

Figure S1. A diagram of the natural history of HIV infection. All movement is in one direction except for enrollment in and dropout from interventions from ART.

Ordinary Differential Equations:

The model simulates a population from ages 0 to 59 in five-year age-groups, capturing vertical transmission and aging. The system of ODE's describes the states $X_{a,r}^{g,d,v}(t)$ with the following indices:

- g refers to gender

  $g = 0$ for males; $g = 1$ for females

- d refers to disease state defined by CD4 cell count, and treatment and circumcision status

  $d = 0$ for HIV-negative; $d = 1$ for acute infection; $d = 2$ for CD4 >500 cells/μL; $d = 3$ for CD4 500-350 cells/μL; $d = 4$ for CD4 350–200 cells/μL; $d = 5$ for CD4 <200 cells/μL; $d = 6$ for HIV-negative, circumcised, and no PrEP; $d = 7$ for HIV-negative, circumcised, and on PrEP; $d = 8$ for HIV-negative, uncircumcised, and on PrEP; $d = 9$ for HIV-positive on ART

- v refers to disease state defined by viral load

  $v = 0$ for HIV-negative; $v = 1$ for acute infection; $v = 2$ for VL<1,000 copies/mL; $v = 3$ for VL 1,000-10,000 copies/mL; $v = 4$ for VL 10,000-50,000 copies/mL; $v = 5$ for VL>50,000 copies/mL; $v = 6$ for HIV-positive and on ART

- a refers to age group

  $a = 0$ for ages 0 to 4; $a = 1$ for ages 5 to 9; … ; $a = 11$ for ages 55 to 59

- r refers to sexual risk group defined by number of sexual partnerships per year

  $r = 0$ for low risk; $r = 1$ for medium risk; $r = 2$ for high risk

The ODEs for the nine disease states are:

$$\frac{dX_{a,r}^{g,0,0}(t)}{dt} = b_{r,0}^{g,0}(t) + \sigma_{a,r}^{g,0} X_{a,r}^{g,7,0}(t) - \left(\mu_a^g + \lambda_{a,r}^{g,0}(t) + \pi_{a,r}^{g,0,0}(t)\right) X_{a,r}^{g,0,0}(t)$$

$$\frac{dX_{a,r}^{g,1,v}(t)}{dt} = b_{r,0}^{g,1}(t) + \lambda_{a,r}^{g,0} X_{a,r}^{g,0,0}(t) + \psi_0 \lambda_{a,r}^{1,0}(t) X_{a,r}^{1,6,v}(t) + \psi_0 \psi_1 \lambda_{a,r}^{1,1}(t) X_{a,r}^{1,7,0}(t)$$
$$+ \psi_1 \lambda_{a,r}^{g,1}(t) X_{a,r}^{g,9,6}(t) + \sigma_{a,r}^{g,1}(t) X_{a,r}^{g,9,6} - \left(\mu_a^g + \alpha_a^{g,1} + \nu_1 + \pi_{a,r}^{g,1,v}(t)\right) X_{a,r}^{g,1,v}(t)$$

$$\frac{dX_{a,r}^{g,2,v}(t)}{dt} = (\nu_1 + \omega_{v-1}) X_{a,r}^{g,1,v}(t) + \sigma_{a,r}^{g,2} X_{a,r}^{g,9,6}(t)$$
$$- \left(\mu_a^g + \alpha_a^{g,2} + \nu_2 + \omega_v + \pi_{a,r}^{g,2,v}(t)\right) X_{a,r}^{g,2,v}(t)$$

$$\frac{dX_{a,r}^{g,3,v}(t)}{dt} = (\nu_2 + \omega_{v-1})X_{a,r}^{g,2,v}(t) + \sigma_{a,r}^{g,3}X_{a,r}^{g,9,6}(t)$$

$$- \left(\mu_a^g + \alpha_a^{g,3} + \nu_3 + \omega_v + \pi_{a,r}^{g,3,v}(t)\right)X_{a,r}^{g,3,v}(t)$$

$$\frac{dX_{a,r}^{g,4,v}(t)}{dt} = (\nu_3 + \omega_{v-1})X_{a,r}^{g,3,v}(t) + \sigma_{a,r}^{g,4}X_{a,r}^{g,9,6}(t)$$

$$- \left(\mu_a^g + \alpha_a^{g,4} + \nu_4 + \omega_v + \pi_{a,r}^{g,4,v}(t)\right)X_{a,r}^{g,4,v}(t)$$

$$\frac{dX_{a,r}^{g,5,v}(t)}{dt} = (\nu_4 + \omega_{v-1})X_{a,r}^{g,4,v}(t) + \sigma_{a,r}^{g,5}X_{a,r}^{g,9,6}(t)$$

$$- \left(\mu_a^g + \alpha_a^{g,5} + \nu_5 + \omega_v + \pi_{a,r}^{g,5,v}(t)\right)X_{a,r}^{g,5,v}(t)$$

$$\frac{dX_{a,r}^{g,6,0}(t)}{dt} = b_{r,1}^{g,0}(t) + \sigma_{a,r}^{g,0}X_{a,r}^{g,6,0}(t) - \left(\mu_a^g + \psi_0\lambda_{a,r}^{g,0}(t) + \pi_{a,r}^{g,0,0}(t)\right)X_{a,r}^{g,6,0}(t)$$

$$\frac{dX_{a,r}^{g,7,0}(t)}{dt} = \pi_{a,r}^{g,0,0}(t)X_{a,r}^{g,5,0}(t) - \left(\sigma_{a,r}^{g,0} + \mu_a^g + \psi_0\psi_1\lambda_{a,r}^{g,1}(t)\right)X_{a,r}^{g,7,0}(t)$$

$$\frac{dX_{a,r}^{g,8,0}(t)}{dt} = \pi_{a,r}^{g,0,0}(t)X_{a,r}^{g,0,0}(t) - \left(\sigma_{a,r}^{g,0} + \mu_a^g + \psi_1\lambda_{a,r}^{g,1}(t)\right)X_{a,r}^{g,8,0}(t)$$

$$\frac{dX_{a,r}^{g,9,6}(t)}{dt} = \sum_{v=1}^{5}\sum_{d=1}^{5}[\pi_{a,r}^{g,d,v}(t)X_{a,r}^{g,d,v}(t) - \left(\sigma_{a,r}^{g,d} + \mu_a^g\right)X_{a,r}^{g,9,6}(t)]$$

The equation variables are:

$b_{r,c}^{g,d}(t)$	The number of births that are HIV-negative ($d = 0$), HIV-positive ($d = 1$), uncircumcised ($c = 0$), or circumcised ($c = 1$)
$\sigma_{a,r}^{g,d}$	The dropout rate from PrEP ($d = 0$) or ART ($d = 1, \dots, 5$)
μ_a^g	The background mortality
$\lambda_{a,r}^{g,d}(t)$	The force of infection for HIV-negative persons on PrEP ($d = 1$) or off PrEP ($d = 0$)
$\pi_{a,r}^{g,d,v}(t)$	The coverage of PrEP ($d = 0$), ART ($d = 1, \dots, 5$), circumcision ($d = 6$), condom use among HIV-negative persons ($d = 7$), condom use among PrEP users ($d = 8$), and condom use among ART users ($d = 9$)
$\alpha_a^{g,d}$	The HIV-associated mortality

ν_d	The rate of progressing from CD4 state d to $d+1$
ω_d	The rate of progressing from VL state v to $v+1$
ψ_d	The reduction in HIV transmission due to circumcision ($d=0$), PrEP ($d=1$), ART ($d=2$), or condom use ($d=3$)

Demography:

At each iteration, the force of infection and the number of births are calculated and then used to evaluate the ODEs along with mortality and disease progression. The numbers of incident infections, HIV-related deaths, and individuals entering CD4≤200 cells/µL are also calculated to determine QALYs.

Births:

The number of births, $b_{r,c}^{g,d}(t)$, determines how many newborns enter the population of gender g, disease state d, sexual risk group r, and circumcision status c ($c=0$ for uncircumcised; $c=1$ for circumcised males). For simplicity, we assume only neonatal circumcision (the circumcision level is increased over time such that 30% of males are circumcised by 2013, as currently observed in Kampala and shown in Figure S2 [2]), that infected births enter the acute stage, and that women age 15–49 give birth. Fertility rates are stratified by age and stage of disease. Births from uninfected mothers, $bS(t)$, and from HIV-positive mothers, $bI(t)$, are:

$$bS(t) = \sum_{a=3}^{9}\sum_{r=0}^{2}\left[\gamma_a^0 X_{a,r}^{1,0,0}(t) + \gamma_a^9 X_{a,r}^{1,9,6}(t)\right]$$

$$bI(t) = \sum_{a=3}^{9}\sum_{r=0}^{2}\sum_{d=1}^{5}\sum_{v=1}^{5}\gamma_a^d X_{a,r}^{1,d,v}(t) + \sum_{a=3}^{9}\sum_{r=0}^{2}\gamma_a^9 X_{a,r}^{1,9,6}(t)$$

HIV-negative births for uncircumcised males, $b_{r,0}^{0,0}(t)$, are:

$$b_{r,0}^{0,0}(t) = 0.5 * \phi_{0,r}^{0,0} * \left(bS(t) + \left(1-\eta(t)\right)bI(t)\right) * (1 - \pi_{0,r}^{1,5}(t))$$

HIV-negative births for circumcised males, $b_{r,1}^{0,0}(t)$, are:

$$b_{r,1}^{0,0}(t) = 0.5 * \phi_{0,r}^{0,0} * \left(bS(t) + \left(1 - \eta(t)\right)bI(t)\right) * \pi_{0,r}^{1,5}(t)$$

HIV-negative births for females, $b_{r,0}^{1,0}(t)$, are:

$$b_{r,1}^{1,0}(t) = 0.5 * \phi_{0,r}^{1,0} * \left(bS(t) + \left(1 - \eta(t)\right)bI(t)\right)$$

HIV-positive births for males and females, $b_{r,0}^{g,1}(t)$, are:

$$b_{r,0}^{g,1}(t) = 0.5 * \phi_{0,r}^{g,0} * \eta(t)bI(t)$$

The equation variables are:

$\phi_{a,r}^{g,d}$	The proportion of individuals in age a, gender g, and treatment status d ($d = 0$, no treatment; $d = 1$, PrEP; $d = 2$, ART) that is born into sexual risk group r
$\eta(t)$	The proportion of births from HIV-positive females that result in vertical transmission
$\pi_{0,r}^{1,5}(t)$	The proportion of HIV-negative males that is circumcised at birth
γ_a^d	The annual fertility rate for females by age and disease state

Each birth is multiplied by 0.5 given an assumed gender ratio at birth of 1:1.

Figure S2. Proportion of males circumcised over time.

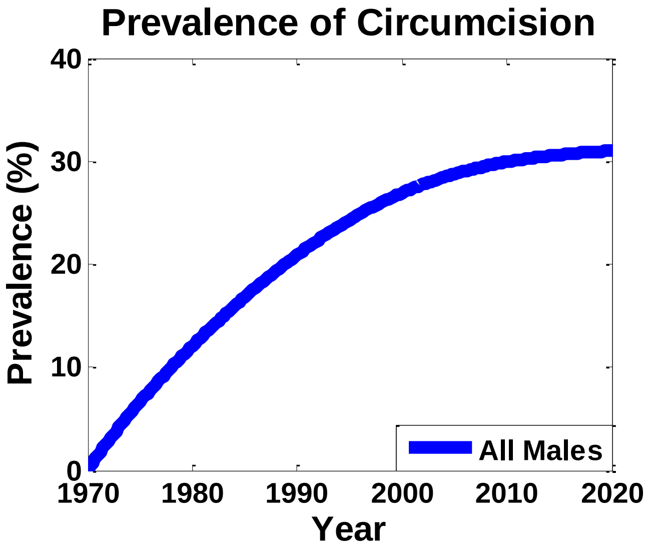

Mortality:

People leave the population due to death or aging past age 59. Mortality is represented by mortality caused by HIV, $\alpha_a^{g,d}$, and all other background mortality, μ_a^g. Mortality caused by HIV varies by stage of disease and age (individuals 0 to 4 years old and 50 to 59 years old are assumed to have elevated risks of death), and individuals on ART are assumed to have no

disease-induced mortality [3, 4]. The background mortality rate is estimated to be the population mortality rate in 1990, prior to the generalized HIV epidemic.

Disease Transmission:

Disease transmission is governed by the force of infection, $\lambda_{a,r}^{g,d}(t)$, which determines the number of people who are infected at each time-step.

$$\lambda_{a,r}^{g,d}(t) = \sum_{a'=0}^{11}\sum_{r'=0}^{2}\left[c_{g,a,r}^{*a',r'}(t)\rho_{g,a,r}^{a',r'}(t) * \frac{(\sum_{v'=1}^{5}\psi_3 X_{a',r'}^{g',d',v'}(t)\beta^{g,r,v'}) + \psi_3\psi_4 X_{a',r'}^{g',9,6}(t)\beta^{g,r,6}}{\sum_{v'=0}^{6} X_{a',r'}^{g',d',v'}(t)}\right]$$

The equation variables are:

$c_{g,a,r}^{*a',r'}(t)$	The number of partners from age a' and sexual risk group r' that an individual has per year
$\rho_{g,a,r}^{a',r'}(t)$	The mixing matrix which describes the distribution of partners from each age and sexual risk group
$\beta^{g,r,v'}$	The probability of HIV transmission per partnership between an HIV-positive person of stage v' and HIV-negative person of risk group r

The overall force of infection for a specific age-group is the sum of the risk of acquiring HIV from all possible partners.

Mixing Matrix:

Using methods similar to other models, the mixing matrix, $\rho_{g,a,r}^{a',r'}(t)$, describes patterns of sexual contact by calculating the proportion of one's sexual partners that come from a specific age and sexual-risk group [5].

$$\rho_{g,a,r}^{a',r'}(t) = \left[\epsilon_a \frac{\sum_{r'=0}^{2}(c_{a',r'}^{g'}\sum_{d'=0}^{9}\sum_{v'=0}^{6} X_{a',r'}^{g',d',v'}(t))}{\sum_{a'=0}^{11}\sum_{r'=0}^{2}(c_{a',r'}^{g'}\sum_{d'=0}^{9}\sum_{v'=0}^{6} X_{a',r'}^{g',d',v'}(t))} + (1-\epsilon_a)\delta_a^{a'}\right]$$

$$* \left[\epsilon_r \frac{c_{a',r'}^{g'}\sum_{d'=0}^{9}\sum_{v'=0}^{6} X_{a',r'}^{g',d',v'}(t)}{\sum_{r'=0}^{2}(c_{a',r'}^{g'}\sum_{d'=0}^{9}\sum_{v'=0}^{6} X_{a',r'}^{g',d',v'}(t))} + (1-\epsilon_r)\delta_r^{r'}\right]$$

where	$\delta_r^{r'}$	$= 1.0$	If $r = r'$
		$= 0.0$	If $r \neq r'$
Before 2005:	$\delta_a^{a'}$	$= 0.3$	If $a = a'$
		$= 0.7$	If $a = a' + 1$ (for males)<br>If $a = a' - 1$ (for females)
		$= 0.0$	Otherwise
After 2005:	$\delta_a^{a'}$	$= 0.7$	If $a = a'$
		$= 0.3$	If $a = a' + 1$ (for males)<br>If $a = a' - 1$ (for females)
		$= 0.0$	Otherwise

Mixing patterns vary between random and assortative, as determined by the parameter ϵ. Random mixing ($\epsilon = 1$) is mixing proportional to the relative sizes of all compartments and this method is consistent for both random mixing by risk and by age. However, assortative mixing ($\epsilon = 0$) is among groups with similar characteristics and differs for mixing by risk and age. Assortative mixing by risk ($\epsilon_r = 0$) is defined by the identity matrix $\delta_r^{r'}$, whereas assortative mixing by age ($\epsilon_a = 0$) is defined by an off-diagonal matrix $\delta_a^{a'}$. The off-diagonal pattern results in females of age a being more likely to form partnerships with males of age $a = a' - 1$, which is consistent with reports of such age discrepancies in sub-Saharan Africa [6, 7]. Although this off-diagonal method results in some age groups having fewer than 100% of their partnerships, those age-groups are $a = 0$ and $a = 11$, which contribute relatively little to overall HIV transmission. We assume that this tendency for age-gaps diminishes in 2005. Furthermore, ϵ_a and ϵ_r shift from random to assortative over the course of the simulation, given the consistent government campaigns against risky sexual behavior [2].

Per-Partnership Probability of Transmission:

The per-partnership probability of transmission, $\beta^{g,r,d'}$, depends on the sexual risk group of the HIV-negative partner and the disease state of the HIV-positive partner. The probabilities of transmission per partnership are:

$$\beta^{0,r,v'} = 1 - (1 - \chi^{v'})^{A_r^0} \qquad \text{For male HIV-negative partners}$$
$$\beta^{1,r,v'} = \left(1 - (1 - \chi^{v'})^{A_r^1}\right) \qquad \text{For female HIV-negative partners}$$

$\chi^{g,d'}$ is the per-act probability of transmission for an HIV-positive partner of HIV stage d', and the exponent, A_r^g, is the number of coital acts based on the HIV-negative partner's sexual risk group and gender.

Rate of Partner Change:

Data on sexual behavior and specifically, sexual contact rates, $c_{a,r}^g$, are often subject to biases leading to contact rate data that, when assuming solely heterosexual contact, are inconsistent between males and females [8]. We account for this variability by using an adjusted contact rate, $c^{*a'r'}_{g,a,r}(t)$, which equilibrates the reported number of sexual partners by males and females [5]. The adjusted contact rate can be male- or female-driven, as determined by the parameter θ, where $\theta = 1$ for male-driven, $\theta = 0$ for female-driven, and $\theta = 0.5$ when compromised equally. We assume $\theta = 0.5$ given the lack of data to assume otherwise. The adjusted contact rate for females is:

$$c_{1,a,r}^{*a',r'}(t) = c_{a,r}^1 B_{a,r}^{a',r'}(t)^{-(1-\theta)}$$

For males, the adjusted contact rate is:

$$c_{0,a,r}^{*a',r'}(t) = c_{a,r}^0 B_{a,r}^{a',r'}(t)^{\theta}$$

The discrepancy between the two populations, $B_{a,r}^{a'r'}(t)$, is defined as:

$$B_{a,r}^{a',r'}(t) = \frac{c_{a,r}^{0}\rho_{0,a,r}^{a',r'}(t) * \sum_{d=0}^{9}\sum_{v=0}^{6} X_{a,r}^{0,d,v}(t)}{c_{a,r}^{1}\rho_{1,a,r}^{a',r'}(t) * \sum_{d=0}^{9}\sum_{v=0}^{6} X_{a,r}^{1,d,v}(t)}$$

Population Aging

To age the population, one-fifth of each compartment enters the next age group of corresponding gender, sexual risk, and disease state. When individuals age, they also change sexual risk; therefore, they redistribute to a set sexual-risk profile, $\phi_{a,r}^{g,d}$, that varies by age, gender, and treatment status. All compartments, except for the youngest and oldest age-groups, experience influx from the prior age and efflux into the next age. The 0 to 4 age-group only receives influx through births while the 55 to 59 age-group exits the population rather than entering the next age. Therefore, each state has a second ODE that occurs at each time step:

$$\frac{dX_{0,r}^{g,d}(t)}{dt} = -\frac{1}{5}X_{0,r}^{g,d}(t) \qquad \text{For } a = 0$$

$$\frac{dX_{a,r}^{g,d}(t)}{dt} = -\frac{1}{5}X_{a,r}^{g,d}(t) + \frac{1}{5}\sum_{r=0}^{2} X_{a-1,r}^{g,d}(t)\,\phi_{a-1,r}^{g,d} \qquad \text{For } a \neq 0$$

II. Interventions

Enrollment is represented by $\pi_{a,r}^{g,d,v}(t)$, while dropout is represented by $\sigma_{a,r}^{g,d}$.

ART Treatment Enrollment:

Coverage of ART treatment for HIV-positive persons increases from 0% in 2000 to 44% for persons with CD4 ≤200 cells/μL in 2003 as previously observed in Uganda [9], then to 50% coverage for persons with CD4 ≤350 cells/μL in 2013 as observed in the Home HTC study [10]. Treatment coverage then changes depending on the specified coverage and scale-up period. ART treatment is assumed to reduce the likelihood of HIV transmission by 96% as suggested by recent studies ($\psi_2 = 0.96$), and persons on ART are expected to have the same life expectancy as HIV-negative persons of similar age and gender, and thus, are assumed not to be subject to

HIV-associated mortality [4, 11-14]. The annual drop-out rate is 6%, which is equally likely for all individuals regardless of their HIV state prior to treatment. Individuals who drop out of ART return to the infected stages at the same proportion with which they enrolled.

Circumcision:

This model includes a background level of circumcision of 30% as currently observed in Kampala [2]. Several studies show that circumcised males have a 60% ($\psi_0 = 0.6$) lower risk of acquiring HIV, but are not at a reduced risk of transmitting HIV [15-17]. Therefore, the model does no track the circumcision status of HIV-positive persons. The ODE for HIV-negative circumcised males is:

$$\frac{dX_{a,r}^{0,6,0}(t)}{dt} = b_{r,1}^{0,0}(t) + \sigma_{a,r}^{0,0} X_{a,r}^{1,7,0}(t) - \left(\mu_a^0 + (1-\psi_0)\lambda_{a,r}^{0,0}(t) - \pi_{a,r}^{0,6,0}(t)\right) X_{a,r}^{0,6,0}(t)$$

Other models have studied the impact of circumcision in-depth to include wound healing periods and sexual activity [18, 19]. However, this model assumes that circumcision is instantaneous.

III. Epidemiological Parameters

Table S1. Risk distribution by age for males and females, calibrated to fit age-specific HIV incidence and prevalence data.

Age Cohort	Male Risk Distribution			Female Risk Distribution			Reference
	Low-Risk	Moderate-Risk	High-Risk	Low-Risk	Moderate-Risk	High-Risk	
**0 – 4**	0.999	0.0005	0.0005	0.999	0.0005	0.0005	Calibrated to fit data
**5 – 9**	0.999	0.0005	0.0005	0.999	0.0005	0.0005	
**10 – 14**	0.98	0.015	0.005	0.98	0.015	0.005	
**15 – 19**	0.80	0.17	0.03	0.85	0.13	0.02	
**20 – 24**	0.78	0.20	0.02	0.75	0.22	0.03	
**25 – 29**	0.65	0.28	0.07	0.68	0.27	0.05	
**30 – 34**	0.55	0.35	0.10	0.72	0.23	0.05	
**35 – 39**	0.65	0.28	0.07	0.75	0.20	0.05	
**40 – 44**	0.71	0.23	0.06	0.79	0.16	0.05	
**45 – 49**	0.78	0.17	0.05	0.80	0.16	0.04	
**50 – 54**	0.87	0.09	0.04	0.90	0.08	0.02	
**55 – 59**	0.95	0.04	0.01	0.95	0.04	0.01	

Table S2. Background mortality in Uganda

Age Cohort	Background Mortality		Reference
	Male	Female	
**0 – 4**	.06	.06	UNICEF [20]
**5 – 9**	.00674	.00608	WHO [21]
**10 – 14**	.00480	.00430	
**15 – 19**	.00471	.00432	
**20 – 24**	.00850	.00895	
**25 – 29**	.01500	.01548	
**30 – 34**	.02092	.01654	
**35 – 39**	.02742	.01770	
**40 – 44**	.02892	.01710	
**45 – 49**	.02890	.02423	
**50 – 54**	.02604	.02352	
**55 – 59**	.04593	.01735	

Table S3. Fertility rate as a proportion of the female population 15 to 49 years old and varying by disease state. Females on ART are assumed to have equal fertility to HIV-negative females.

Age Cohort	Fertility Rate (per year)					Reference
	**Uninfected** RR=1	**Acute** RR=1	**>350** RR=0.42	**200-350** RR=0.42	**<200** RR=0.59	
**0 – 4**	0	0	0	0	0	Anderson *et al.*, Ross *et al.* [7, 22]
**5 – 9**	0	0	0	0	0	
**10 – 14**	0	0	0	0	0	
**15 – 19**	0.175	0.175	0.0788	0.0473	0.0332	
**20 – 24**	0.313	0.313	0.1409	0.0845	0.0595	
**25 – 29**	0.324	0.324	0.1458	0.0875	0.0616	
**30 – 34**	0.271	0.271	0.1221	0.0732	0.0515	
**35 – 39**	0.201	0.201	0.0905	0.0543	0.0382	
**40 – 44**	0.125	0.125	0.0563	0.0338	0.0238	
**45 – 49**	0.053	0.053	0.0239	0.0143	0.0101	
**50 – 54**	0	0	0	0	0	
**55 – 59**	0	0	0	0	0	

Table S4. HIV-associated mortality estimates are from observational studies of untreated HIV-positive persons. Persons age 0 to 4 and older than 50 are assumed to have greater mortality as observed.

Age Cohort	HIV Mortality				Reference
	**Acute**	**CD4>350**	**CD4 200 to 350**	**CD4<200**	
**0 – 4**	0.47	0.47	0.47	0.47	Newell *et al.* [23]
**5 – 49**	0.01	0.05	0.08	0.27	Badri *et al.* [24]
**50 – 59**	0.02	0.10	0.16	0.54	Adler *et al.* [3]

Table S5. The probability of transmission per coital act by HIV stage and treatment status.

Baseline	Increase in transmission probability by HIV stage	Reference

Transmission Probability							
	**Acute**	**VL ≤1,000**	**VL 1,000-10,000**	**VL 10,000-50,000**	**VL >50,000**	**ART**	
0.0006	26	1	5.8	6.9	11.9	0.04	Quinn *et al.*, Boily *et al.* [25, 26]

Table S6. The duration of time in each CD4 and viral load stage by gender

CD4 Transition	Acute	CD4>500	500-350	350-200
Time for Males (years)	0.25	1.71	1.05	4.71
Time for Females (years)	0.25	1.94	1.35	6.71
**Viral Load Transition**	**Acute**	**VL≤1,000**	**1,000-10,000**	**10,000-50,000**
Time for Males (years)	0.25	3.44	1.45	3.04
Time for Females (years)	0.25	3.06	2.27	5.45

Table S7. The number of coital acts per partnership by gender and sexual risk group, calibrated to fit age-specific HIV incidence and prevalence data.

Gender	Coital Acts per Partnership			Reference
	**Low-Risk**	**Moderate-Risk**	**High-Risk**	
**Male**	99	33	3.3	Calibrated to fit data
**Female**	77	22	3.3	

Table S8. The mixing parameter varies from random ($\boldsymbol{\epsilon = 1}$) to assortative ($\boldsymbol{\epsilon = 0}$), calibrated to fit age-specific HIV incidence and prevalence data.

Year	Force of Infection Mixing	Reference

	ϵ_a (age)	ϵ_r (sexual risk)	
**Before 1998**	0.7	0.7	
**2003**	0.5	0.5	Calibrated to fit data
**After 2010**	0.1	0.1	

IV. References

1. International Data Base: Uganda 1980. In: U.S. Census Bureau.
2. International UMoHaI. 2011 Uganda AIDS Indicator Survey: Key Findings. In. Maryland, USA: MOH and ICF International; 2012.
3. Adler WH, Baskar PV, Chrest FJ, Dorsey-Cooper B, Winchurch RA, Nagel JE. HIV infection and aging: mechanisms to explain the accelerated rate of progression in the older patient. *Mech Ageing Dev* 1997,**96**:137-155.
4. Mills EJ, Bakanda C, Birungi J, Mwesigwa R, Chan K, Ford N, *et al.* Mortality by baseline CD4 cell count among HIV patients initiating antiretroviral therapy: evidence from a large cohort in Uganda. *AIDS* 2011,**25**:851-855.
5. Garnett GP, Gregson S. Monitoring the course of the HIV-1 epidemic: The influence of patterns of fertility on HIV-1 prevalence estimates. *Mathematical Population Studies* 2000,**8**:251-277.
6. Ott MQ, Bärnighausen T, Tanser F, Lurie MN, Newell ML. Age-gaps in sexual partnerships: seeing beyond 'sugar daddies'. *AIDS* 2011,**25**:861-863.
7. Anderson R, May R, Ng T, Rowley J. Age-Dependent Choice of Sexual Partners and the Transmission Dynamics of HIV in Sub-Saharan Africa. *Phil. Trans. R. Soc. London. B* 1992,**336**:135 - 155.
8. Burington B, Hughes JP, Whittington WL, Stoner B, Garnett G, Aral SO, *et al.* Estimating duration in partnership studies: issues, methods and examples. *Sex Transm Infect* 2010,**86**:84-89.
9. Combat HIV/AIDS, malaria, and other diseases. In. Geneva: UNDP; 2013.
10. Barnabas RV, van Rooyen H, Tumwesigye E. Initiation of antiretroviral therapy and viral suppression after home HIV testing and counselling in KwaZulu-Natal, South Africa, and Mbarara district, Uganda: a prospective, observational intervention study. In. Lancet HIV; 2014.
11. Attia S, Egger M, Müller M, Zwahlen M, Low N. Sexual transmission of HIV according to viral load and antiretroviral therapy: systematic review and meta-analysis. *AIDS* 2009,**23**:1397-1404.
12. Cohen MS, Chen YQ, McCauley M, Gamble T, Hosseinipour MC, Kumarasamy N, *et al.* Prevention of HIV-1 infection with early antiretroviral therapy. *N Engl J Med* 2011,**365**:493-505.
13. Donnell D, Baeten JM, Kiarie J, Thomas KK, Stevens W, Cohen CR, *et al.* Heterosexual HIV-1 transmission after initiation of antiretroviral therapy: a prospective cohort analysis. *Lancet* 2010,**375**:2092-2098.
14. Jahn A, Floyd S, Crampin AC, Mwaungulu F, Mvula H, Munthali F, *et al.* Population-level effect of HIV on adult mortality and early evidence of reversal after introduction of antiretroviral therapy in Malawi. *Lancet* 2008,**371**:1603-1611.
15. Auvert B, Taljaard D, Lagarde E, Sobngwi-Tambekou J, Sitta R, Puren A. Randomized, controlled intervention trial of male circumcision for reduction of HIV infection risk: the ANRS 1265 Trial. *PLoS Med* 2005,**2**:e298.
16. Gray RH, Kigozi G, Serwadda D, Makumbi F, Watya S, Nalugoda F, *et al.* Male circumcision for HIV prevention in men in Rakai, Uganda: a randomised trial. *Lancet* 2007,**369**:657-666.

17. Weiss HA, Quigley MA, Hayes RJ. Male circumcision and risk of HIV infection in sub-Saharan Africa: a systematic review and meta-analysis. *AIDS* 2000,**14**:2361-2370.
18. Hallett TB, Singh K, Smith JA, White RG, Abu-Raddad LJ, Garnett GP. Understanding the impact of male circumcision interventions on the spread of HIV in southern Africa. *PLoS One* 2008,**3**:e2212.
19. Williams BG, Lloyd-Smith JO, Gouws E, Hankins C, Getz WM, Hargrove J*, et al.* The potential impact of male circumcision on HIV in Sub-Saharan Africa. *PLoS Med* 2006,**3**:e262.
20. South Africa Basic Indicators. In: United Nations Children's Education Fund.
21. Life Tables for WHO Member States: Uganda. In. Geneva: WHO.
22. Ross A, Van der Paal L, Lubega R, Mayanja BN, Shafer LA, Whitworth J. HIV-1 disease progression and fertility: the incidence of recognized pregnancy and pregnancy outcome in Uganda. *AIDS* 2004,**18**:799-804.
23. Newell ML, Coovadia H, Cortina-Borja M, Rollins N, Gaillard P, Dabis F*, et al.* Mortality of infected and uninfected infants born to HIV-infected mothers in Africa: a pooled analysis. *Lancet* 2004,**364**:1236-1243.
24. Badri M, Lawn SD, Wood R. Short-term risk of AIDS or death in people infected with HIV-1 before antiretroviral therapy in South Africa: a longitudinal study. *Lancet* 2006,**368**:1254-1259.
25. Quinn TC, Wawer MJ, Sewankambo N, Serwadda D, Li C, Wabwire-Mangen F*, et al.* Viral load and heterosexual transmission of human immunodeficiency virus type 1. Rakai Project Study Group. *N Engl J Med* 2000,**342**:921-929.
26. Boily MC, Baggaley RF, Wang L, Masse B, White RG, Hayes RJ*, et al.* Heterosexual risk of HIV-1 infection per sexual act: systematic review and meta-analysis of observational studies. *Lancet Infect Dis* 2009,**9**:118-129.
